# pCRP-mCRP Dissociation Mechanisms as Potential Targets for the Development of Small-Molecule Anti-Inflammatory Chemotherapeutics

**DOI:** 10.3389/fimmu.2018.01089

**Published:** 2018-05-28

**Authors:** Vittorio Caprio, Lina Badimon, Mario Di Napoli, Wen-Hui Fang, Glenn R. Ferris, Baoqiang Guo, Rocco S. Iemma, Donghui Liu, Yasmin Zeinolabediny, Mark Slevin

**Affiliations:** ^1^Faculty of Science and Engineering, School of Healthcare Science, Manchester Metropolitan University, Manchester, United Kingdom; ^2^Hospital de la Santa Creu I Sant Pau, IIB Sant Pau, Barcelona, Spain; ^3^Neurological Service, Ospedale San Camillo de Lellis, Rieti, Italy; ^4^Institute of Dementia and Neurological Aging, Weifang Medical University, Weifang, China; ^5^University of Medicine and Pharmacy, Targu Mures, Romania

**Keywords:** CRP, inflammation, chemotherapy, phospholipid, phospholipase

## Abstract

Circulating C-reactive protein (CRP) is a key acute-phase protein and one of the main clinical biomarkers for inflammation and infection. CRP is an important upstream mediator of inflammation and is associated with the onset of a number of important disease states including cardiovascular disease and neurodegenerative disorders such as Alzheimer’s disease. This pentraxin exerts pro-inflammatory properties *via* dissociation of the pentamer (pCRP) to a monomeric form (mCRP). This dissociation is induced by binding of pCRP to cell surface phosphocholine residues exposed by the action of phospholipase A_2_ (PLA_2_). Given the association of CRP with the onset of a range of serious disease states this CRP dissociation process is a tempting drug target for the development of novel small-molecule therapeutics. This review will discuss potential targets for chemotherapeutic intervention elucidated during studies of CRP-mediated inflammation and provide an up-to-date summary of the development of small molecules, not only targeted directly at inhibiting conversion of pCRP to mCRP, but also those developed for activity against PLA_2_, given the key role of this enzyme in the activation of CRP.

## Introduction

Pentameric C-reactive protein (p-CRP) is a pentraxin, composed of five identical subunits, linked by van der Waals and H-bonding, each weighing around 23 kDa with, what is described as, a jelly role shape with the subunits arranged around a central, hydrophobic pore. The pentamer presents two faces, each distinguished by their binding capabilities. Thus, the A face (effector face) binds to globular head groups of compliment c1q and Fcγ cell surface receptors on leukocytes while the B (binding) face exhibits one binding site per subunit which undergoes Ca^2+^-mediated binding with phosphocholine moieties exposed on lipid membranes (1). pCRP is synthesized in the liver and is freely circulating. While normally present at negligible levels, plasma concentrations rise 6–12 h after acute inflammatory insult to 1,000-fold levels after 24–48 h, focused at sites of inflammation (2, 3). As a result, CRP is used as a biomarker for inflammation and infection. It was long thought that pCRP was a direct mediator of inflammation leading to upregulation of endothelial cellular adhesion molecules, activation of the compliment system, phagocytosis, and release of a range of inflammatory signaling proteins (4, 5). However, it has recently been shown that the dissociation into the monomeric form, mCRP, is the key pro-inflammatory event (6). Further work has shown that this event is localized to sites of inflammation and mCRP plays an important role in the pathogenesis of inflammation interacting with endothelial cells, neutrophils, macrophages, and platelets (7). mCRP, rather than pCRP, induces upregulation of IL-8, MCP-1, E-selectin, ICAM-1, and VCAM-1 in endothelial cells resulting in increased adhesion of neutrophils (8). These studies reveal that this process is mediated *via* p38 MAPK signaling. Interestingly, recent work indicates that the interaction with endothelial cells is initiated *via* binding to lipid rafts rather than receptors, such as FcγRs on the cell surface (9, 10). CRP is a ligand for LOX-1 which mediates the entry of oxidized low-density lipoprotein (ox-LDL) across the endothelium (11). Furthermore, mCRP is implicated in the uptake of ox-LDL by macrophages leading to foam cell formation (12). mCRP can also activate monocytes to adhere to endothelia and transmigrate—a process mediated *via* binding with integrin receptors (13, 14). High local levels of mCRP have been detected in the myocardium of patients suffering from acute coronary syndrome (15) and the choroids obtained from donors at high risk of developing age-related macular degeneration (16). Furthermore, it has been shown there is an accumulation of mCRP in pertinent brain regions, arising from poststroke inflammation (17) and evidence that this observation explains the known link between ischemic stroke and onset of AD (18). In addition, Aβ plaques have been demonstrated to cause dissociation of pCRP to mCRP leading to a buildup of the latter in cortical tissue of AD patients (19).

**Figure 1:**
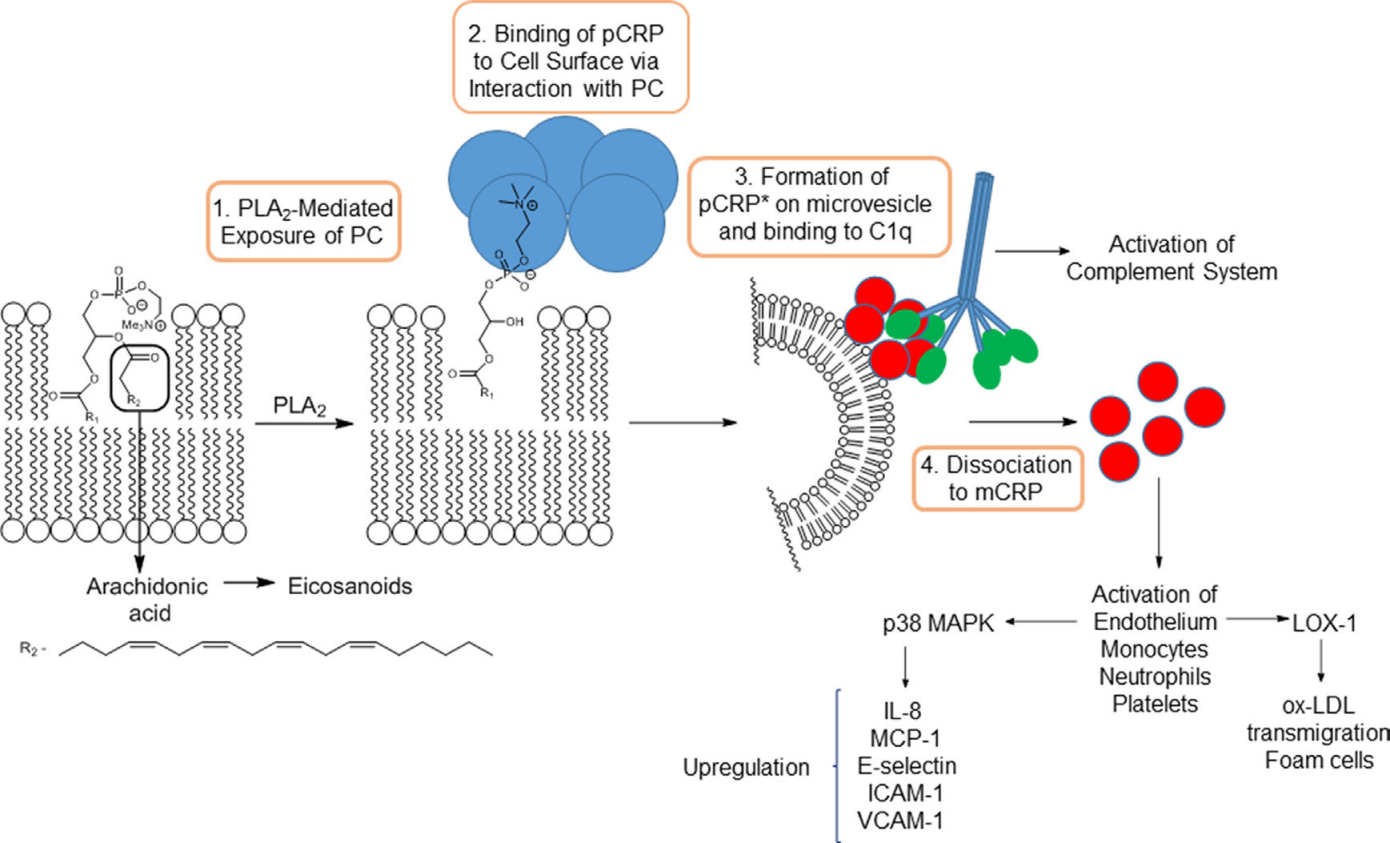
Action of PLA_2_ on arachidonic acid-containing phospholipids and subsequent mechanism of dissociation of pCRP to mCRP.

The dissociation of pCRP to mCRP has now been delineated in some detail. The dissociation is mediated by binding of pCRP subunits to phosphocholine residues of lysophosphotidylcholines (LPC) exposed on cell membranes (Figure 1). LPC is generated by the action of pro-inflammatory phospholipase (PLA_2_) enzymes acting on cell surface lysophospholipids. This link between PLA_2_ and CRP-mediated inflammation is backed up by the 6–12 h delay observed between inflammatory insult and onset of high levels of CRP. Furthermore, CRP formation is prevented by pre-incubation of monocytes with ONO-RS-82, a well-known inhibitor of PLA_2_ enzymes (20). Dissociation is also mediated *via* interaction with phosphocholine present on the surface of activated platelets, which acts to localize mCRP generation to areas of inflammation such as atherosclerotic plaques (13). Localized dissociation may also arise from binding of pCRP to lysophosphocholine residues exposed on the surface of ox-LDL, by lipoprotein-associated PLA_2_ (Lp-PLA_2_) (11). The most recent studies have provided a more detailed mechanism of dissociation (21). Binding of pCRP on activated monocytes, in addition to docking with phosphocholine, also involves interactions between hydrophobic regions of the pentamer and lipid rafts on the cell surface. The protein is then released onto microvesicles and undergoes a conformational change to an activated pentamer designated pCRP*. This moiety, while still pentameric, exists in a more open form and undergoes binding with a globular head group of complement C1q, which inserts into the central cavity forcing the subunits of the pentamer further apart to ultimately cause dissociation to mCRP.

## Chemotherapeutic Targets in CRP Dissociation

The clear link between pCRP-mCRP dissociation and the onset/mediation of inflammation indicates that inhibition of this process is, potentially, a valuable chemotherapeutic strategy for the treatment of a range of conditions associated with the inflammatory response. A number of key stages, from initial exposure of cell surface phosphocholine residues to mCRP-mediated activation of monocytes/platelets/endothelia potentially provide an opportunity for chemotherapeutic inhibition. However, an understanding of these various processes at the molecular level is an important prerequisite for the development of small molecules abrogation. Fortunately, investigations have provided information on amino acid–ligand interactions by *in silico* modeling, site-directed mutagenesis studies, and X-ray crystallographic information. For instance, an X-ray crystal structure of pCRP bound to phosphocholine reveals key amino acids involved in ligand binding (1). Significantly, a hydrophobic cavity is shown to exist, adjacent to the binding region, providing a potential blueprint for the design of inhibitors of pCRP–phosphocholine binding. Furthermore, an X-ray crystal structure of a CRP dissociation inhibitor, 1,6-bis(phosphocholine)-hexane, a drug discussed further below, bound to the active of two CRP pentamers has also been determined (22). A crystal structure of the globular head group of C1q has been solved, and the information used to provide a model for the interaction of this domain with p-CRP and to postulate amino acid residue interactions involved in complement-pentamer binding (23). Site-directed mutagenesis studies have also been directed toward identifying the key CRP–C1q interactions (24). mCRP-mediated activation of monocytes via binding to integrins αvβ3 and α4β1 has also been simulated by *in silico* modeling yielding identification of potential binding sites (14). Significantly, this study, while predicting favorable mCRP-integrin binding, indicates significant steric interactions in pCRP-integrin models of binding. The identification of lipid raft interactions as key to mCRP binding to a range of targets, including endothelia, *via* cholesterol binding sequence (9, 10) offers an additional target for small-molecule intervention-although this interaction has not been studied at similar levels of details to some of those discussed above.

These studies provide information that can be used to develop small-molecule agents to inhibit the interaction between pCRP and phosphocholine, complement C1q-induced dissociation to mCRP and subsequent activation of monocytes. However, to date, the only stage which has which has been perturbed by small-molecule agents is the initial binding of pCRP to phosphocholine, to be discussed herein. Nevertheless, an important stage of CRP activation is exposure of phosphocholine residues on cell surfaces by PLA_2_ and the action of this enzyme has been linked to CRP-mediated inflammation (20). A large number of small molecules have been developed to inhibit phospholipase activity although only a small number have been shown to lower levels of mCRP (20). However, the use of PLA_2_ inhibitors to treat neuroinflammation, *via* suppression of pro-inflammatory lysophospholipid formation, has been postulated (25) and, given the clear links between mCRP formation and lysophospholipid exposure, further implicates the use of PLA_2_ inhibitors to prevent CRP dissociation. Thus, this review will focus on summarizing work in this area.

## Small-Molecule Inhibitors of Phospholipase A_2_

Among the various subgroups within the phospholipase A_2_ superfamily, secreted phospholipase A_2_ (sPLA_2_), cytosolic phospholipase A_2_ (cPLA_2_), and lipoprotein-associated phospholipase A_2_ (LpPLA_2_) have been the most popular targets for the development of inhibitors. The development of small molecules against the PLA_2_ family has been extensively reviewed and this mini review will seek to provide a brief, up-to-date overview of only the most successful drug candidates against s-, c-, and LpPLA_2_ (26).

All PLA_2_ enzymes catalyze the hydrolysis of phospholipids at cell membranes or the surface of lipoproteins, to produce free fatty acids and exposing lysophospholipids, including LPC, on the cell surface (Figure 1). The former may include arachidonic acid, which is converted to inflammatory-mediating eicosanoids, indicating a dual pro-inflammatory role for PLA_2_ enzymes.

**Figure 2:**
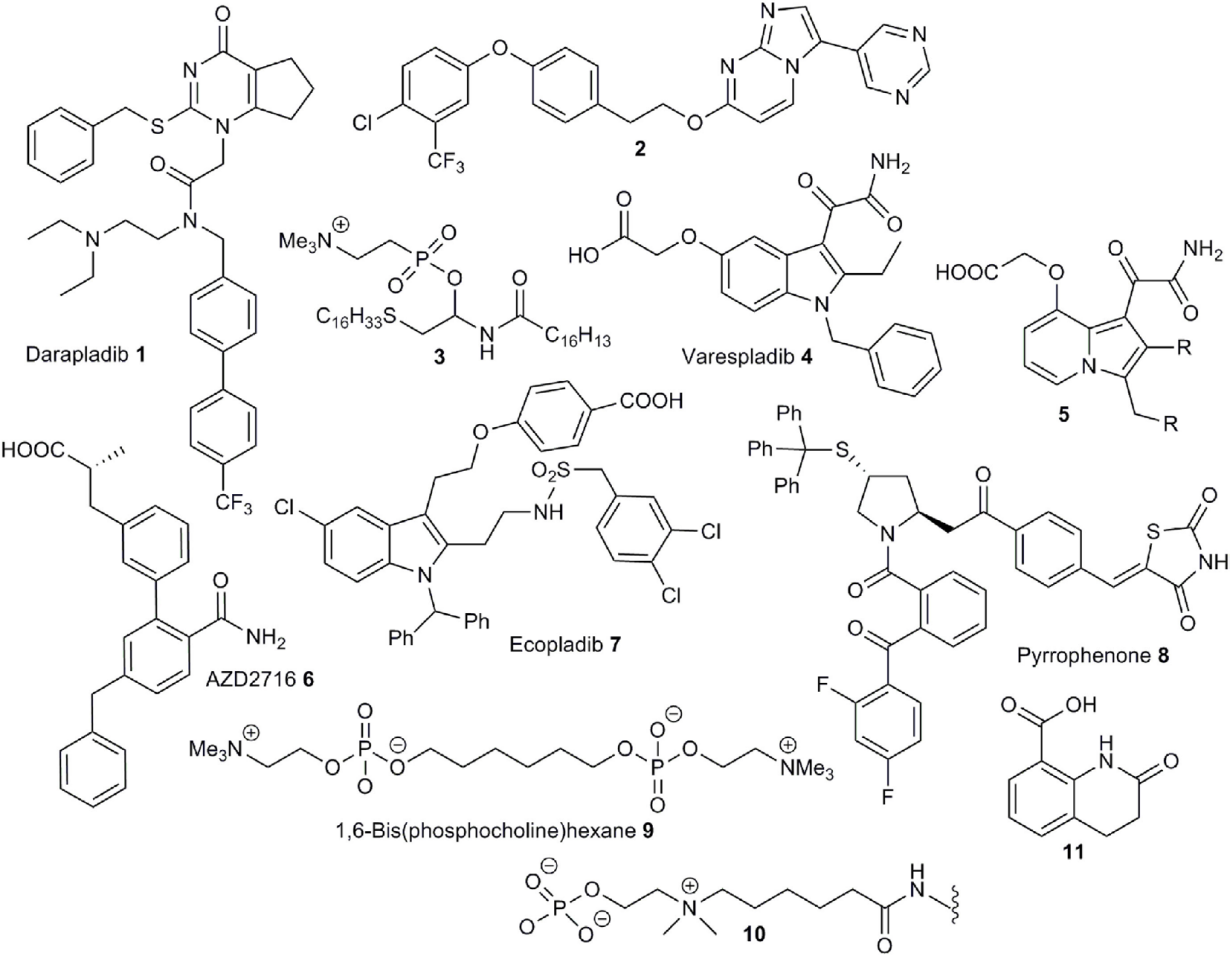
Example structures of anti-PLA_2_ drugs and small-molecule binders to CRP.

Lipoprotein-associated phospholipase (LpPLA_2_) hydrolyzes oxidized phospholipids present on the surface of ox-LDL producing pro-inflammatory oxidized fatty acids and lysophospholipids (27). A plausible link between LpPLA_2_ activity and CRP activation is supported by the detection of CRP/ox-LDL complexes in the plasma of atherosclerosis patients (28). The central role of this enzyme in the development of inflammation has led to its use as a predictive biomarker for the onset of atherosclerosis (29). A diversity of structures have been discovered to exhibit LpPLA_2_ inhibition (30–35). The most successful drugs against LpPLA_2_ are pyrimidin-4-ones of the darapladib class **1** (Figure 2) (36) discovered by modification of lead compounds unearthed by high throughput screening programs at GSK (37–39). A range of analogs, based on the darapladib motif have been studied but do not display improved activity (40, 41), although some imidazopyrimidine derivatives, such as **2**, do exhibit improved bioavailability (42). Unfortunately, darapladib failed Phase III clinical trials due to a failure to alleviate the risk of cardiovascular death or stroke in coronary heart disease patients (43, 44).

Secretory phospholipase A_2_ (sPLA_2_) is an extracellular phospholipase catalyzing the hydrolysis of phospholipids at cell surfaces. The association of this enzyme with the development of inflammatory conditions, and even some cancers, has driven the development of a number of small-molecule inhibitors (45). Unsurprisingly, phospholipid derivatives do serve as inhibitors given the natural substrates for this enzyme class (46–48). For instance, the thioether analog **3** is a potent inhibitor (49). The phosphocholine group has been successfully substituted with a carboxylic acid moiety, which appears to function as a bioisostere for this group, to provide compounds with excellent anti-sPLA_2_ activity (50) and substitution of the trimethylammonium group with an amide provides more permeable compounds with some inhibitory properties (51). The most successful molecules against sPLA_2_ are those based on an indole-3-acetamide structure. Structure-activity studies based around this central motif (52, 53), aided by an X-ray crystal structure of recombinant enzyme co-complexed with a lead compound (54, 55) led to the development of the 3-glyoxamide derivative varespladib **4** (56). Unfortunately, as with darapladib, varespladib failed to negotiate Phase III trials due to lack of efficacy (57). Significantly, indole-based compound, closely related to **4**, are also potent inhibitors of group X sPLA_2_, mammalian phospholipases, which are particularly active pro-inflammatory members of this enzyme family (58). Furthermore, X-ray structures of these inhibitors bound to the active site have been obtained (59). Related indolizines such as **5** also exhibit potent anti-sPLA_2_ activity (60) and the importance of a central heterocyclic aromatic core to this activity is reflected by the use of this information to develop potent inhibitors based around pyrazole fragments (61). This concept was later expanded to the study of amide-functionalized aromatic fragments leading to the development of the preclinical candidate AZD2716 **6** (62). Compound **6** exhibits better oral bioavailability than varapladib, which requires deployment as a methyl ester prodrug.

In contrast to sPLA_2_, cPLA_2_ functions is an intracellular enzyme and specifically interacts with arachidonyl phospholipids and is thus especially responsible for the formation of pro-inflammatory arachidonic acid in addition to lysophospholipids. This enzyme has been identified as a key mediator of inflammation leading to a range of disease states (63). A range of relatively simple compounds have been found to act as potent inhibitors of activity. The design of these is largely based on mimicking the arachidonoyl phosphonate structure and a knowledge of the serine-based mechanism of phospholipid hydrolysis. While a hydrophobic chain or aromatic group acts as a replacement for the arachidonate moiety, an activated ketone serves to disrupt serine hydrolysis and, as is the case with sPLA_2_ inhibitors, a carboxylate is an effective surrogate for the phosphonate group (64). The early, anthranilic acid-based broad spectrum, PLA_2_ inhibitors such as *N*-(p-amylcinnamoyl)anthranilic acid (ACA) and ONO-RS-82 (65), widely used as tools to probe PLA_2_ activity, partially fit this model for inhibitor design as does the selective cPLA_2_ inhibitor arachidonyltrifluoromethylketone (AA-COCF_3_) (66). A design strategy based on phospholipid binding has also led to the development of linear 2-oxoamides (67) and 2-oxoesters (68) linked *via* nitrogen or oxygen, respectively, to aliphatic carboxylic acid group, and bis-aryloxypropanones, where both aliphatic groups around a central carbonyl group have been replaced with aromatic moieties (64). The disubstituted propanones serve as useful motifs for inhibitor design and replacement of one aromatic group with a thiazole (69), or suitably substituted indoles (70), have yielded cPLA_2_ inhibitors with good activity. The indole moiety has been identified as a suitable substitute for the arachidonate section of the phospholipid substrate, and this strategy has led to the development of the ecopladib **7** class of cPLA_2_ inhibitors (71). Structural modification of **7** led to the development of the closely related efipladib (72) and giripladib (73). The latter compound was advanced to Phase II trials but terminated at this stage. High-throughput screening approaches have also led to the discovery of potent cPLA_2_ inhibitors. Compound library screening yielded two fragments—a pyrrolidine and a thiazolidinylidene, combination of which provided a series of compounds, such as pyrrophenone **8**, with very high inhibitory activity (74, 75).

## Small-Molecule Inhibitors of PCRP Dissociation

The only small molecule demonstrated to inhibit dissociation of pCRP to mCRP is the bis-phosphocholine dimer 1,6-bis(phosphocholine)-hexane (bis(PC)-H) **9** (22). The design of this compound utilized a similar strategy used in the development of drugs targeted toward serum amyloid P component (SAP) which act to crosslink two SAP molecules and is based on the utilization of moieties chemically similar to phosphocholine head groups that bind to the same active site to disrupt LPC-mediated CRP activation. Crucially, a X-ray crystal structure of the pCRP-bis(PC)-H drug complex was obtained revealing binding of five drug molecules to phosphocholine binding sites to link two pentamers. This interaction abrogates binding of pCRP to known ligands such as LDL and blocks CRP-mediated complement C1q activation. Additionally, bis(PC)-H was demonstrated to reduce CRP-mediated effects in rat models. Despite demonstration of some clinical efficacy in animal models bis(PC)-H suffers from a low half-life, low CRP affinity and other suboptimal pharmacokinetic parameters.

While bis(PC)-H is the only small molecule that has been demonstrated to effectively disrupt CRP dissociation, *via* direct binding, other compounds have been shown to undergo chemical interactions with this pentamer and thus provide potential blueprints for the future design of inhibitors. For instance, a polypeptide conjugated with the phosphocholine linker **10** is a high-affinity binder to CRP demonstrating that phosphocholine mimics, free from the cell surface, can effectively interact with the active sites of the pentamer (76, 77). Furthermore, effective binding of **10** indicates that the CRP active sites may tolerate phosphocholine analogs with larger, extended alkyl chains as has been indicated previously by the X-ray crystal structure of the CRP-phosphocholine complex (1). Further work in this area has revealed that conjugates bearing heterocycles such as **11** also function as high-affinity binders (78). The dissimilarity between **11** and phosphocholine, and the competition experiments, indicates that there are alternate regions on the surface of CRP that may provide targets for future inhibitor design. Finally, rosuvastatin inhibits CRP-mediated inflammation in rat models expressing human CRP (79). As this treatment does not reduce circulating levels of CRP, effects are not solely down to inhibition of gene expression but rather to inhibition of CRP-mediated pathways. Direct binding to CRP has not been established however.

## Conclusion

The dissociation of pCRP to mCRP is clearly an important event in the onset of inflammatory processes implicated in major disease states and inhibition is thus an important chemotherapeutic goal. It is surprising that only one compound has been developed that successfully inhibits dissociation *via* direct binding to CRP and the lack of follow-up studies Thus, the use of PLA_2_ inhibitors to indirectly affect dissociation is potentially the most promising current strategy given the range of structures available and proven efficacy. However, few have been demonstrated to exert effects on mCRP formation, and the failure of all anti-PLA_2_ drugs evaluated in advanced trials is a cause for concern. Nevertheless, studies have revealed a range of well-characterized potential chemotherapeutic targets for inhibition of CRP dissociation and, given the recent discoveries of non-natural small-molecule binders to CRP, it is anticipated that the search for drugs that abrogate CRP-mediated inflammation will be a rich area of research in the future.

## Author Contributions

VC prepared a large part of the body of text and figures. LB, MN, W-HF, GF, BG, RI, DL, YZ, and MS assisted in manuscript preparation and provided critical evaluation of the work.

## Conflict of Interest Statement

The authors declare that the research was conducted in the absence of any commercial or financial relationships that could be construed as a potential conflict of interest.
